# Disparities in the Diagnosis and Treatment of Bile Duct Cancer in People with Disabilities: A National Cohort Study in South Korea

**DOI:** 10.3390/ijerph192416625

**Published:** 2022-12-10

**Authors:** Seon Mee Park, So Young Kim, Kyoung Eun Yeob, Dong Wook Shin, Joung-Ho Han, Jong Heon Park, Jong Hyock Park

**Affiliations:** 1 Department of Internal Medicine, College of Medicine, Chungbuk National University, Cheongju 28644, Korea; 2 Division of Gastroenterology, Department of Internal Medicine, Chungbuk National University Hospital, Cheongju 28644, Korea; 3 Department of Public Health and Preventive Medicine, Chungbuk National University Hospital, Cheongju 28644, Korea; 4 Institute of Health & Science Convergence, Chungbuk National University, Cheongju 28644, Korea; 5 Supportive Care Center/Department of Family Medicine, Samsung Medical Center, Seoul 06351, Korea; 6 Department of Digital Health, SAIHST, Sungkyunkwan University, Seoul 03063, Korea; 7 Big Data Steering Department, National Health Insurance Service, Wonju 26464, Korea

**Keywords:** bile duct cancer, disability, stage, treatment, survival

## Abstract

We aimed to evaluate the impacts of disability on the diagnosis, treatment, and prognosis of bile duct cancer (BDC) according to the severity and type of disability. Patients diagnosed with BDC were selected from an age- and sex-matched population (1:3 ratio) with or without disabilities from the National Disability Database, the Korean Central Cancer Registry, and the Korean National Health Insurance claims database. The cohort included 15,065 patients with BDC, with a significantly lower rate in those with severe disabilities than in people without or with mild disabilities (110.6 vs. 136.5 vs. 147.6 per 105 persons, respectively). People with severe disabilities were diagnosed with BDC at an earlier age but were less likely to undergo surgery (adjusted odds ratio (aOR) = 0.52, 95% confidence interval (CI): 0.45–0.61) or chemotherapy (aOR = 0.76, 95% CI: 0.61–0.95) compared to those without disabilities. This trend was more evident in patients with mental disabilities. The overall and cancer-specific mortality rates were higher in patients (especially women) with disabilities than in those without. There needs systemic approach to ensure equal access to quality cancer care for people with disabilities.

## 1. Introduction

Cancer diagnosis and treatment are determined by socio-economic factors as well as tumor biological factors [1,2]. Low-income people are often diagnosed with cancer at a later stage, are less likely to receive standard therapy, and have shorter survival times [2]. In South Korea, disability-related health disparities are reported in terms of health factors such as insufficient physical activity, being underweight or overweight [3], and lower participation in health screening programs [4]. People with disabilities are less likely to undergo staging work-up and intensive treatment for lung [5] and breast cancers [6]. They are also more likely to die prematurely because of cardiovascular disease or cancer [7]. However, disparities vary depending on the cancer, disability type, severity, and living area [5,6,8]. People with severe disabilities; communication, brain, or mental disabilities; or those living in rural areas have less access to cancer screening [9]. However, people with mild disabilities are more likely to use health services than those without disabilities [10]. To the best of our knowledge, there have been no studies on the effects of disabilities on the clinical characteristics, management, and prognosis of bile duct cancer (BDC).

BDC is more prevalent in Asian countries than in Western countries [11]. In South Korea, BDC was the ninth-most common cancer and sixth cause of cancer-related mortality in 2017 [12]. Most patients present with advanced disease and less than 30% are eligible for curative resection. The 5-year survival rate is less than 30% despite improvements in prognosis in recent years [12]. Therefore, early diagnosis and intensive treatment are necessary to improve the prognosis of BDC [13].

In South Korea, all people are covered by universal health insurance. The coinsurance for cancer work-up and treatment is only 5% of the total medical costs, with a maximum copay for low-income people of only approximately USD 1000 since 2016. In addition, Korea has a national disability registration system, which defines disability type and severity according to preset criteria and medical diagnosis. These are optimal conditions for examining disparities in BDC related to disabilities. Using the linked administrative database in Korea, we investigated the potential disparities in the diagnosis, treatment, and survival of patients diagnosed with BDC between people with and without disabilities.

## 2. Materials and Methods

### 2.1. Data Sources and Case Selection

This study obtained data from the Korean National Disability Registry (KNDR) [14], the Korean Central Cancer Registry (KCCR), and the Korean National Health Insurance (KNHI) claims database. The KNDR contains information about disability type and severity, and included 93.8% of the total population with disabilities in 2011. The KCCR, a nationwide government-sponsored cancer registry, includes information regarding age and date at diagnosis, cancer site, and Surveillance, Epidemiology, and End Results (SEER) summary stage, and included 97.2% of cancer cases in 2017 [12]. Among SEER staging categories (localized, regional, metastatic, and unknown), the unknown stage is assigned if the primary cancer site is unknown or sufficient evidence is not available to adequately assign a stage. The KNHI provides universal health insurance to all Koreans, and the database contains information regarding patient health insurance premium, residential area, comorbid diseases, diagnosis, imaging and laboratory results, treatment, and mortality. Information from the KNDR was merged with data from the KCCR and KNHI claims database for the period 2002–2015 using personal identification numbers.

We identified 2,776,450 persons with disabilities from 2009 to 2013 in an age- and sex-matched population (1:3 ratio) that also included 8,329,350 people without disabilities. Fifteen disability types listed in the KNDR were categorized as physical, communication, brain, mental, or affecting the major internal organs (Supplementary Table S1). Physical disability was the most common type (1,436,219 people, 51.7%), followed by communication disability (585,986 people, 21.1%), brain disability (307,026 people, 11.1%), mental disability (307,026, 11.1%), and major internal organ disability (139,499 people, 5.0%). Disability severity is graded from 1 (very severe) to 6 (very mild) based on functional losses and clinical impairment that are determined by a specialist. In this study, disability severity was also classified as severe (grades 1–3) or mild (grades 4–6), with 1,091,794 people (39.3%) in the severe group and 1,684,656 people (60.7%) in the mild group. Among the entire cohort, 67.4% were <65 years old and 32.6% were ≥65 years old; 58.5% were men and 41.5% were women. People with mental disabilities had the youngest median age (33.6 years). The KNHI premium was used to estimate household income because it is calculated based on income, property, and automobile taxes for each household; household income was categorized as: below the poverty line (lowest) and quartiles I, II, III, and IV (highest), as defined by the KNHI [2]. The proportion of people below the poverty line was 7.1% in the entire cohort, 16.9% in the disabled group, and 3.9% in the non-disabled group. Approximately half of the people with mental disabilities (45.8%) had incomes below the poverty line (Supplementary Table S2).

**Figure 1:**
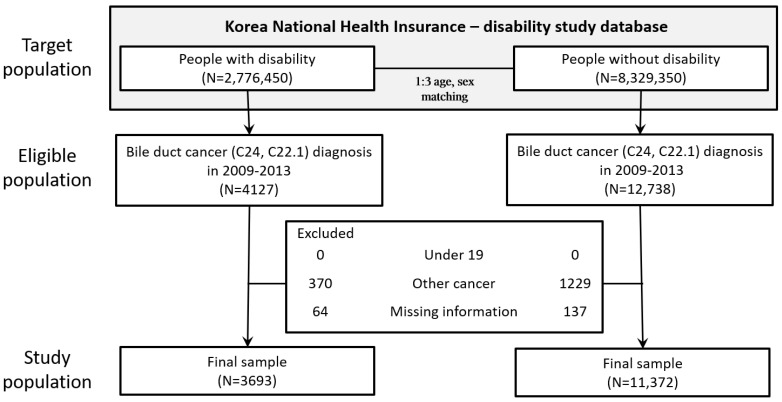
CONSORT diagram.

We subsequently identified 16,865 patients with diagnosed BDC (intrahepatic cholangiocarcinoma C22.1 and extrahepatic cholangiocarcinoma C24.0–24.9) and excluded patients who were <19 years old (n = 0), had no information regarding health insurance premiums (n = 201), or had a diagnosis of other non-thyroid cancer (n = 1599). Finally, 3693 patients with disabilities and 11,372 patients without disabilities at BDC diagnosis were enrolled in the study population (Figure 1). The study protocol was approved by the Institutional Review Board of Chungbuk National University (CBNU-201708-BM-501-01; Cheongju, South Korea). 

For each patient, information was collected about sex, age, severity and type of disability, Charlson comorbidity index (CCI), and presence of comorbidities including hypertension (HTN), diabetes mellitus (DM), coronary heart disease (CHD), stroke, chronic obstructive pulmonary disease (COPD), and cholelithiasis. Information on socioeconomic factors such as income level and location of residence, including whether it was an endemic area for clonorchiasis, was collected. We also selected data regarding BDC such as date at diagnosis, SEER summary stage (local, regional, distant, and unknown), specific treatment (surgery, radiotherapy, and chemotherapy), and overall and cancer-specific mortality.

### 2.2. Outcomes and Statistical Analyses

Descriptive analyses were performed to determine the distributions of patients with and without disabilities according to age, sex, and income level in patients with BDC. The chi-square test was used to compare categorical variables. The relative probability of receiving surgery and chemotherapy were calculated using logistic regression analyses and adjusted for age, sex, CCI, income, location of residence, and cancer stage. All patients were followed until death or 31 December 2017. The survival outcomes and related risk factors were determined using the Kaplan–Meier method with the log-rank test and multivariate Cox proportional hazards regression analysis. To confirm the assumption of proportionality, we used the graphic method by plotting log hazard estimates against observation periods. Each covariate that was used for adjustment had a Schoenfeld residual indicating that the proportional-hazard assumption was fulfilled (*p* > 0.1 for all covariates). All statistical analyses were performed using SAS software (version 9.4; SAS Institute, Inc., Cary, NC, USA) and *p* ≤ 0.05 was considered to denote statistical significance.

## 3. Results

### 3.1. Diagnosis of BDC

**Figure 2:**
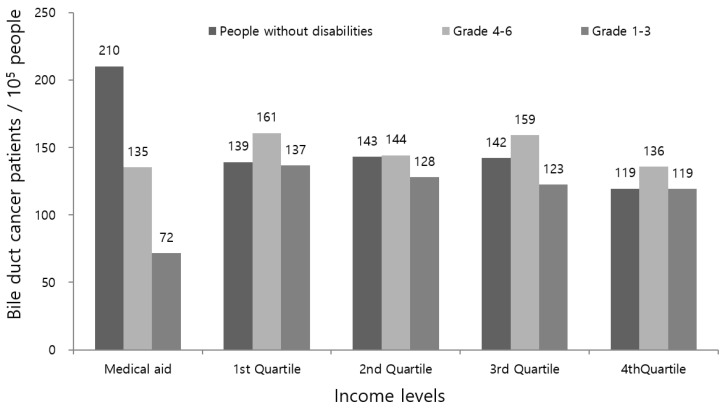
Comparison of income level structure in patients with BDC between those with or without disabilities (household income level was categorized as: below the poverty line (medical aid, lowest income level) and quartiles I, II, III, and IV (highest); disability severity was also classified as severe (grades 1–3) or mild (grades 4–6)).

The diagnostic rate of BDC was slightly lower in people with disabilities than in those without (133.0 vs. 136.5 per 105 people, respectively). In addition, the rates were significantly lower in people with severe disabilities compared to those with mild disabilities (110.6 vs. 147.6 per 105 people, respectively). Among disability types, patients with mental disabilities had the lowest diagnostic rate, followed by those with internal organ disabilities. The mean age at BDC diagnosis was younger in severely disabled people than in mildly or non-disabled people (68.9 vs. 69.8 vs. 70.3 years, respectively). People with mental or internal organ disabilities were diagnosed with BDC at an earlier age than were people with other types of disabilities. Severely disabled people with BDC had a higher CCI than mildly disabled or non-disabled people (2.3 vs. 1.9 vs. 1.4, respectively). Patients with brain or internal organ disabilities had a higher CCI and a higher prevalence of HTN, DM, CHD, stroke, and COPD compared to patients with other types of disabilities. The proportion of BDC patients among individuals with incomes below the poverty line was higher in the disabled group than in the non-disabled group, especially in those with severe disabilities and mental disabilities. The diagnostic rate of BDC was lowest in people with severe disabilities and incomes below the poverty line (71.3 per 10^5^ people; Figure 2). More patients with BDC in the disabled group than in the non-disabled group lived in rural (*p* < 0.0001) and endemic areas for clonorchiasis (*p* = 0.035) (Table 1).

### 3.2. Analysis of Treatment Behaviors

There were no differences in SEER stages in patients with or without disabilities. However, a higher proportion of localized or unknown stages was observed in the severely disabled group compared to the mildly disabled group for all types of disabilities. Patients with brain or mental disabilities had a higher unknown stage of BDC compared to patients with other types of disabilities (Table 2).

Among the 15,065 patients with BDC, 5318 (35.3%) received surgery. The proportion of patients who received surgery was slightly lower in those with disabilities than in those without (32.7% vs. 36.2%; adjusted odds ratio (aOR) = 0.83, 95% confidence interval (CI): 0.76–0.91) after adjusting for age, sex, CCI, income, location of residence, and cancer stage. This disparity was more significant in the severe group (24.3% vs. 36.2%; aOR = 0.52, 95% CI: 0.45–0.61), with the surgery rate gradually increasing from grade 1 to grade 3. Among disability types, patients with severe grades of mental (aOR = 0.31, 95% CI: 0.17–0.57), brain (aOR = 0.34, 95% CI: 0.24–0.47), or internal organ (aOR = 0.38, 95% CI: 0.24–0.60) disabilities had the lowest rates of surgery. Among the 15,065 patients with BDC, 1,579 (10.5%) received chemotherapy. There were no differences in chemotherapy rates between patients with disabilities and those without (10.0% vs. 10.6%; aOR = 0.90, 95% CI: 0.79–1.03). However, patients with severe disabilities received less chemotherapy than did those without disabilities (9.2% vs. 10.6%; aOR = 0.75, 95% CI: 0.61–0.95). Among the various disability types, severe brain (aOR = 0.58, 95% CI: 0.37–0.90) were associated with lower rates of chemotherapy compared to other disability types (Table 3 and Table 4).

### 3.3. Survival Analysis

**Figure 3:**
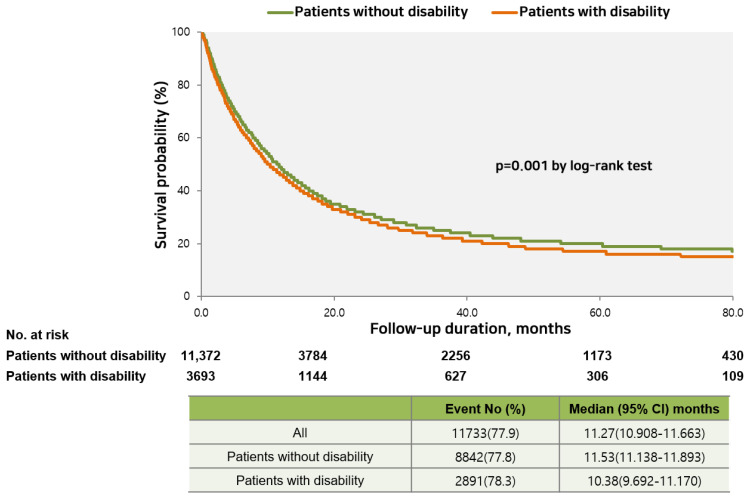
Comparison of cancer-specific survival between patients with and without disabilities.

The median BDC-specific survival time among all patients was 11.3 months; 10.4 and 11.5 months in patients with and without disabilities, respectively. After adjusting for age, sex, income, endemic area, CCI, and SEER stage, we observed poorer BDC-specific mortality in patients with disabilities than in those without (536.1 vs. 486.1 per 1000 persons; adjusted hazard ratio (aHR) = 1.05, 95% CI: 1.01–1.10) (Table 5, Figure 3, and Supplementary Figure S1). Among patients with disabilities, the aHR of overall and BDC-specific mortality increased in patients with severe disabilities from grade 3 to grade 1. Patients with mild disabilities (all types) had a mortality rate similar to that of patients without disabilities (Table 5 and Supplementary Table S3-1).

Among the various disability types, patients with severe brain (aHR = 1.65, 95% CI: 1.29–2.12) or mental disabilities (aHR = 1.65, 95% CI: 1.29–2.12) exhibited poorer BDC-specific mortality. When overall and BDC-specific mortality was analyzed separately in men and women, the overall hazard ratio was slightly higher in men than in women with disabilities (Table 6 and Table 7 and Supplementary Tables S3-2 and S3-3). However, mortality was higher in women with mental disabilities compared to their male counterparts.

## 4. Discussion

This study demonstrated that people with disabilities were less likely to be diagnosed, less likely to receive standard treatment, and had lower BDC survival rates compared to those without disabilities. Evident trends were found in patients with severe disabilities or in those with brain or mental disabilities. People with severe disabilities, mental disabilities, and lower incomes had a lower diagnosis rate of BDC in this study. South Korea operates a medical benefits system that provides almost free basic medical services to people at the lowest income level. Among people without disabilities, the diagnosis rates of BDC were higher in individuals with incomes below the poverty line than in the upper income groups. However, in people with severe disabilities, the lowest diagnosis rate was observed among individuals with incomes below the poverty line. These results demonstrate that the current economic support is not sufficient to overcome barriers to accessing medical services that are faced by low-income, severely disabled people [15]. More comprehensive support is needed in terms of managing physical mobility, provision of care, providing easy-to-read information, and improving the awareness regarding the health problems of disabled people.

The diagnosis rate of BDC also varied according to disability type, with the lowest diagnosis rate observed in people with mental (intellectual or psychological) disabilities. Consistent with a previous report [16], this study revealed that people with mental disabilities had a poorer economic status compared to those with other types of disabilities. Therefore, the poor economic status of patients with mental disabilities may also interfere with BDC diagnosis.

Unknown stages of BDC were more frequently reported for those with severe disabilities or brain or mental disorders. This diagnostic outcome was correlated with a less comprehensive evaluation of the tumor extent. These patients were at the lowest income level, had a higher CCI, or lived in rural areas, which interfered with the proper evaluation of BDC. A previous study in Korea reported that disabilities or comorbidities influence tumor stage at diagnosis [17]. 

The mean age of patients at BDC diagnosis was slightly younger in those with disabilities than in those without, and this trend was prominent in those with severe disabilities. People with disabilities have a higher CCI and were more likely to live in endemic areas for clonorchiasis, which may lead to the development of BDC at an earlier age. Among the various comorbidities, DM was a risk factor for BDC [18] and was more prevalent in disabled people. In addition, patients with mental disabilities were diagnosed at the youngest age, and had a relatively short average life span [19] that could preclude survival until BDC diagnosis. 

The BDC treatment patterns also varied according to disability status. Disabled patients underwent less surgery and chemotherapy compared to those without disabilities. These trends were more prominent in patients with severe disabilities or with brain, mental, or internal organ disabilities. Intensive treatment such as surgical resection and adjuvant or palliative chemotherapy is necessary to achieve a good long-term prognosis in BDC [20]. These results were in line with the results of previous studies on different malignancies [5]. A less comprehensive evaluation of tumor extent and less intensive treatment were associated with a lower survival rate in BDC patients with disabilities than in those without disabilities. 

Patients with mental disabilities may not correctly perceive or express their symptoms, which may also delay diagnosis and treatment. Thus, special efforts are needed to obtain accurate information from these patients and to appropriately communicate the treatment that they should receive. For example, experts recommend that interactions with individuals with intellectual disabilities involve plenty of time, simple words, patience, and repeated explanations [21]. 

In this study, women with severe mental disabilities had a worse prognosis than men despite better overall and cancer-specific mortality. Women usually encounter more disadvantages in life compared to men; thus, when women and men without disabilities are compared, the magnitude of the differences may be lower [16]. Women with disabilities may be more likely than their male counterparts to experience stress due to higher rates of poverty, social isolation, violence, and other forms of victimization as well as chronic health problems [22]. Our results showed that such adverse impacts were exacerbated in female BDC patients with mental disabilities.

In this study, we grouped the 15 types of disabilities (limb, visual, auditory, linguistic, brain, facial, kidney, heart, liver, respiratory system, ostomy, epilepsy, intellectual, autistic, and mental) into the following categories: physical (limb, facial, epilepsy), communication (visual, auditory, linguistic), brain, mental (intellectual, autistic, mental), and internal organ impairment (kidney, heart, liver, lung, intestine). We also classified severity levels into two groups: severe (grades 1–3) and mild (grades 4–6). In this study, disparities were more prominent in those with mental disorders, who were the most disadvantaged for every indicator.

To the best of our knowledge, this is the first study to comprehensively analyze potential disparities in the whole spectrum of BDC patients as they relate to disabilities, including the stage at diagnosis, treatment received, and overall and cancer-specific mortality. The strengths of the study included the large number of participants constituting a representative sample, the inclusion of a wide range of disability types, and accurate disability diagnoses.

However, this study had some limitations. First, the use of population-based registries precluded more detailed analyses regarding the other risk factors of BDC, such as primary sclerosing cholangitis, choledochal cysts, or an anomalous union of the pancreaticobiliary duct. However, these specific risk factors are too rare to be identified in many patients [23]. We evaluated the presence of gallstones and the endemic areas of clonorchiasis, because gallstones [24] and clonorchiasis [25] are major risk factors for BDC in Asian countries. Second, detailed information about treatments, such as surgical pathology or chemotherapy cycles, could not be obtained. Despite these limitations, this is the first study to examine the impacts of disability on the diagnosis and treatment of BDC. 

## 5. Conclusions

In conclusion, this study revealed that Korean patients with disabilities face various barriers in terms of BDC diagnosis and receiving effective treatment. These barriers may prevent patients with disabilities from receiving an early diagnosis, staging work-up, and intensive treatment compared to people without disabilities. Therefore, we suggest that economic support must be combined with social support for people with disabilities, including improved awareness of their disabilities, better provision of general care, better communication strategies, and a more inclusive environment.

## Figures and Tables

**Table 1:** General characteristics of the patients with bile duct cancer (BDC).

Variables *	People without Disability	People with Disability	*p* Value	By Disability Grade		By Disability Type				
				Grade 1–3	Grade 4–6	Physical	Communication	Brain	Mental	Internal Organ
All subject, N	8,329,350	2,776,450		1,091,794	1,684,656	1,436,219	585,986	307,026	307,720	139,499
BDC patients, N	11,372	3693		1207	2486	1953	1046	469	72	153
Incidence (N/10^5^)	136.5	133.0	0.003	110.6	147.6	136.0	178.5	152.8	23.4	109.7
Age, years										
Mean ± SD	70.3 ± 9.2	69.5 ± 9.5	0.0003	68.9 ± 10.2	69.8 ± 9.2	68.4 ± 9.2	72.6 ± 9.1	70.4 ± 8.4	56.5 ± 10.7	66.1 ± 9.4
19–39	27 (0.2)	11 (0.3)		6 (0.5)	5(0.2)	5(0.3)	1(0.1)	1(0.2)	3(4.2)	1(0.7)
40–64	2648 (23.3)	988 (26.8)		351 (29.1)	637(25.6)	588(30.1)	196(18.7)	97(20.7)	49(68.1)	58(37.9)
65–74	4810 (42.3)	1503 (40.7)		482 (39.9)	1021(41.1)	835(42.8)	367(35.1)	214(45.6)	20(27.8)	67(43.8)
75–	3887 (34.2)	1191 (32.3)		368 (30.5)	823(33.1)	525(26.9)	482(46.1)	157(33.5)	0(0)	27(17.7)
SEX (Women)	4203 (37)	1390 (37.6)	0.458	412 (34.1)	978(39.3)	796(40.8)	382(36.5)	150(32)	28(38.9)	34(22.2)
CCI										
Mean ± SD	1.4 ± 1.8	2.0 ± 2.1	<0.0001	2.3 ± 2.3	1.9 ± 2.0	1.8 ± 2.0	1.7 ± 1.9	2.9 ± 2.3	1.2 ± 1.7	3.8 ± 2.5
0	4870 (42.8)	1176 (31.8)		357 (29.6)	819 (32.9)	668 (34.2)	369 (35.3)	88 (18.8)	34 (47.2)	17 (11.1)
1	2356 (20.7)	696 (18.9)		196 (16.2)	500 (20.1)	383 (19.6)	225 (21.5)	60 (12.8)	16 (22.2)	12 (7.8)
2	1637 (14.4)	612 (16.6)		204 (16.9)	408 (16.4)	332 (17.0)	171 (16.4)	71 (15.1)	12 (16.7)	26 (17.0)
≥3	2509 (22.1)	1209 (32.7)		450 (37.3)	759 (30.5)	570 (29.2)	281 (26.9)	250 (53.3)	10 (13.9)	98 (64.1)
Comorbidity										
HTN	5344 (47.0)	1924 (52.1)	<0.0001	615 (51)	1309 (52.7)	994 (50.9)	517 (49.4)	305 (65)	15 (20.8)	93 (60.8)
DM	2334 (20.5)	851 (23)	0.0011	285 (23.6)	566 (22.8)	415 (21.3)	230 (22.0)	149 (31.8)	8 (11.1)	49 (32.0)
CHD	1661 (14.6)	664 (18)	<0.0001	230 (19.1)	434 (17.5)	340 (17.4)	161 (15.4)	98 (20.9)	6 (8.3)	59 (38.6)
Stroke	859 (7.6)	559 (15.1)	<0.0001	271 (22.5)	288 (11.6)	174 (8.9)	125 (12.0)	243 (51.8)	2 (2.8)	15 (9.8)
COPD	1656 (14.6)	699 (18.9)	<0.0001	222 (18.4)	477 (19.2)	364 (18.6)	204 (19.5)	70 (14.9)	11 (15.3)	50 (32.7)
Cholelithiasis	6080 (53.5)	2016 (54.6)	0.234	673 (55.8)	1343 (54)	1045 (53.5)	594 (56.8)	252 (53.7)	42 (58.3)	83 (54.3)
Income										
Medical aid	680 (6.0)	438 (11.9)	<0.0001	220 (18.2)	218 (8.8)	193 (9.9)	116 (11.1)	66 (14.1)	43 (59.7)	20 (13.1)
1st Quartile	2449 (21.5)	796 (21.6)		242 (20.1)	554 (22.3)	449 (23)	214 (20.5)	87 (18.6)	10 (13.9)	36 (23.5)
2nd Quartile	2087 (18.4)	643 (17.4)		201 (16.7)	442 (17.8)	372 (19.1)	155 (14.8)	85 (18.1)	5 (6.9)	26 (17.0)
3rd Quartile	2742 (24.1)	843 (22.8)		242 (20.1)	601 (24.2)	444 (22.7)	253 (24.2)	101 (21.5)	7 (9.7)	38 (24.8)
4th Quartile	3414 (30.0)	973 (26.4)		302 (25)	671 (27)	495 (25.4)	308 (29.5)	130 (27.7)	7 (9.7)	33 (21.6)
Residential area										
Seoul	6269 (55.1)	1836 (49.7)	<0.0001	579 (48)	1257 (50.6)	953 (48.8)	535 (51.2)	246 (52.5)	21 (29.2)	81 (52.9)
City	3326 (29.3)	1211 (32.8)		400 (33.1)	811 (32.6)	654 (33.5)	323 (30.9)	159 (33.9)	31 (43.1)	44 (28.8)
Rural	1777 (15.6)	646 (17.5)		228 (18.9)	418 (16.8)	346 (17.7)	188 (18.0)	64 (13.7)	20 (27.8)	28 (18.3)
Endemic area for clonorchiasis										
Yes	5119 (45.0)	1736 (47)	0.035	633 (52.4)	1324 (53.3)	1010 (51.7)	574 (54.9)	265 (56.5)	30 (41.7)	78 (51.0)
No	6253 (55.0)	1957 (53)		574 (47.6)	1162 (46.7)	943 (48.3)	472 (45.1)	204 (43.5)	42 (58.3)	75 (49.0)

* CCI, Charlson comorbidity index; HTN, hypertension; DM, diabetes mellitus; CHD, coronary heart disease; COPD, chronic obstructive pulmonary disease.

**Table 2:** Disability characteristics in patients with localized, regional, and metastatic bile duct cancer.

SEER Stage	Total	Localized	Regional	Metastatic	Unknown
All subject, N	15,065	4062	5637	3077	2289
By disability					
People without disability	11,372	3046 (26.8)	4291 (37.7)	2321 (20.4)	1714 (15.1)
People with disability	3693	1016 (27.5)	1346 (36.5)	756 (20.5)	575 (15.6)
By disability grade					
Grade 1–3	1207	354 (29.3)	375 (31.1)	250 (20.7)	228 (18.9)
Grade 4–6	2486	662 (26.6)	971 (39.1)	506 (20.4)	347 (14.0)
By disability type					
Physical					
Grade 1–3	365	105 (28.8)	126 (34.5)	80 (21.9)	54 (14.8)
Grade 4–6	1588	412 (25.9)	657 (41.4)	314 (19.8)	205 (12.9)
Communication					
Grade 1–3	298	88 (29.5)	98 (32.9)	57 (19.1)	55 (18.5)
Grade 4–6	748	206 (27.5)	272 (36.4)	160 (21.4)	110 (14.7)
Brain					
Grade 1–3	336	103 (30.7)	89 (26.5)	63 (18.8)	81 (24.1)
Grade 4–6	133	40 (30.1)	33 (24.8)	31 (23.3)	29 (21.8)
Mental					
Grade 1–3	72	15 (20.8)	26 (36.1)	16 (22.2)	15 (20.8)
Grade 4–6					
Internal organ					
Grade 1–3	136	43 (31.6)	36 (26.5)	34 (25.0)	23 (16.9)
Grade 4–6	17	4 (23.5)	9 (52.9)	1 (5.9)	3 (17.7)

**Table 3:** Treatment patterns according to the disability characteristics in patients with bile duct cancer.

Characteristics, no. (%)	Surgery ± Chemotherapy ± Radiotherapy *	Chemotherapy± Radiotherapy **	None (Conservative management only)	*p*-Value
No. of patients	5318 (35.3)	1579 (10.5)	8168 (54.2)	
Disability				
Non-disabled patients	4111 (36.2)	1208 (10.6)	6053 (53.2)	<0.0001
Disabled patients	1207 (32.7)	371 (10.0)	2115 (57.3)	
Disability severity				
Grade 1–3	293 (24.3)	111 (9.2)	803 (66.5)	<0.0001
Grade 4–6	914 (36.8)	260 (10.5)	1312 (52.8)	
Grade 1	26 (14.9)	11 (6.3)	137 (78.7)	<0.0001
Grade 2	108 (23.0)	47 (10.0)	315 (67.0)	
Grade 3	159 (28.2)	53 (9.4)	351 (62.3)	
Grade 4	241 (33.7)	68 (9.5)	407 (56.8)	
Grade 5	358 (37.5)	100 (10.5)	496 (52.0)	
Grade 6	315 (38.6)	92 (11.3)	409 (50.1)	
Disabling conditions				
Physical				
Grade 1–3	121 (33.2)	37 (10.1)	207 (56.7)	<0.0001
Grade 4–6	638 (40.2)	190 (12.0)	760 (47.9)	
Communication				
Grade 1–3	72 (24.2)	22 (7.4)	204 (68.5)	
Grade 4–6	231 (30.9)	57 (7.6)	460 (61.5)	
Brain				
Grade 1–3	54 (16.1)	23 (6.8)	259 (77.1)	
Grade 4–6	35 (26.3)	13 (9.8)	85 (63.9)	
Mental				
Grade 1–3	16 (22.2)	9 (12.5)	47 (65.3)	
Grade 4–6	NA	NA	NA	
Internal organ				
Grade 1–3	30 (22.1)	20 (14.7)	86 (63.2)	
Grade 4–6	10 (58.8)	0 (0)	7 (41.2)	

* Patients only had cancer surgery, or they received chemotherapy or radiation therapy with surgery. ** Patients only had chemotherapy or radiation therapy.

**Table 4:** Factors influencing level of surgery, chemotherapy or conservative management in patient with bile duct cancer.

	Surgery ± Chemotherapy ± Radiotherapy * aOR (95% CI)	Chemotherapy ± Radiotherapy ** aOR (95% CI)	Conservative Management Only aOR (95% CI)
By disability			
People without disabilities	1	1	1
People with disability	0.830 (0.756–0.912)	0.903 (0.791–1.030)	1.242 (1.137–1.358)
By disability grade			
People without disabilities	1	1	1
Grade 1–3	0.523 (0.446–0.614)	0.759 (0.610–0.945)	1.991 (1.721–2.305)
Grade 4–6	1.011 (0.908–1.126)	0.976 (0.840–1.134)	1.002 (0.904–1.110)
Grade 1	0.270 (0.171–0.427)	0.476 (0.251–0.904)	4.168 (2.785–6.238)
Grade 2	0.482 (0.375–0.618)	0.818 (0.588–1.137)	2.057 (1.643–2.575)
Grade 3	0.664 (0.533–0.828)	0.804 (0.594–1.089)	1.583 (1.291–1.941)
Grade 4	0.938 (0.777–1.133)	1.010 (0.770–1.324)	1.056 (0.882–1.263)
Grade 5	1.079 (0.917–1.271)	1.004 (0.800–1.261)	0.932 (0.797–1.090)
Grade 6	0.998 (0.839–1.188)	0.922 (0.726–1.170)	1.043 (0.883–1.232)
By disability type			
People without disabilities	1	1	1
Physical			
Grade 1–3	0.752 (0.579–0.978)	0.816 (0.568–1.172)	1.426 (1.114–1.825)
Grade 4–6	1.066 (0.938–1.212)	1.086 (0.914–1.292)	0.905 (0.799–1.024)
Communication			
Grade 1–3	0.652 (0.479–0.889)	0.753 (0.476–1.193)	1.643 (1.235–2.187)
Grade 4–6	0.935 (0.773–1.130)	0.780 (0.583–1.043)	1.175 (0.982–1.407)
Brain			
Grade 1–3	0.337 (0.243–0.468)	0.575 (0.367–0.901)	3.118 (2.335–4.163)
Grade 4–6	0.692 (0.442–1.084)	0.809 (0.442–1.478)	1.516 (1.008–2.280)
Mental			
Grade 1–3	0.310 (0.168–0.574)	0.703 (0.334–1.483)	3.237 (1.895–5.53)
Grade 4–6	NA	NA	NA
Internal organ			
Grade 1–3	0.384 (0.244–0.603)	1.062 (0.636–1.773)	2.125 (1.437–3.143)
Grade 4–6	1.837 (0.580–5.818)	NA	0.981 (0.313–3.070)

Adjusted for age, sex, Charlson comorbidity index, income, place of residence (metropolitan, city, rural), cancer stage. * Patients only had cancer surgery, or they received chemotherapy or radiation therapy with surgery. ** Patients only had chemotherapy or radiation therapy.

**Table 5:** Cancer-specific mortality in patient with bile duct cancer.

	N	Duration	No. of Death	Rate Per 1000	Crude HR	Adjusted HR
By disability						
People without disabilities	11,372	18,189.09	8842	486.115	1	1
People with disability	3693	5392.79	2891	536.085	1.074 (1.030–1.120)	1.054 (1.010–1.099)
By disability grade						
People without disabilities					1	1
Grade 1–3	1207	1472.29	967	656.800	1.238 (1.158–1.323)	1.228 (1.148–1.313)
Grade 4–6	2486	3920.51	1924	490.753	1.007 (0.958–1.058)	0.985 (0.938–1.035)
Grade 1	174	169.87	142	835.924	1.502 (1.273–1.773)	1.588 (1.344–1.876)
Grade 2	470	541.42	377	696.320	1.269 (1.145–1.407)	1.258 (1.134–1.396)
Grade 3	563	761.00	448	588.698	1.150 (1.046–1.264)	1.126 (1.024–1.239)
Grade 4	716	1061.13	560	527.742	1.059 (0.973–1.154)	1.027 (0.942–1.119)
Grade 5	954	1593.81	715	448.611	0.943 (0.874–1.018)	0.929 (0.861–1.003)
Grade 6	816	1265.57	649	512.811	1.039 (0.959–1.125)	1.018 (0.939–1.102)
By disability type						
People without disabilities					1	1
Physical						
Grade 1–3	365	505.7679589	292	577.34	1.126 (1.002–1.265)	1.184 (1.053–1.331)
Grade 4–6	1588	2660.591877	1201	451.403	0.935 (0.881–0.993)	0.947 (0.891–1.006)
Communication						
Grade 1–3	298	352.02	244	693.14	1.273 (1.121–1.446)	1.156 (1.018–1.314)
Grade 4–6	748	1042.85	602	577.265	1.157 (1.065–1.256)	1.067 (0.982–1.159)
Brain						
Grade 1–3	336	346.75	273	787.313	1.450 (1.285–1.635)	1.427 (1.264–1.611)
Grade 4–6	133	183.54	106	577.547	1.163 (0.960–1.408)	1.008 (0.831–1.222)
Mental						
Grade 1–3	72	88.75	57	642.281	1.213 (0.935–1.574)	1.566 (1.203–2.040)
Grade 4–6	–	–	–	–	–	–
Internal organ						
Grade 1–3	136	179.01	101	564.226	1.065 (0.875–1.296)	0.993 (0.815–1.210)
Grade 4–6	17	33.53	15	447.316	0.970 (0.584–1.609)	1.013 (0.610–1.683)

Adjusted for age, sex, income, endemic area, Charlson comorbidity index, seer stage.

**Table 6:** Cancer-specific mortality in male patients diagnosed with bile duct cancer.

	N	Duration	No. of Death	Rate Per 1000	Crude HR	Adjusted HR
By disability						
People without disabilities	7169	11,593.37	5547	478.463	1	1
People with disability	2303	3328.22	1806	542.632	1.102 (1.045–1.162)	1.074 (1.017–1.133)
By disability grade						
People without disabilities					1	1
Grade 1–3	795	995.35	628	630.931	1.220 (1.123–1.325)	1.209 (1.111–1.315)
Grade 4–6	1508	2332.87	1178	504.958	1.048 (0.984–1.116)	1.016 (0.953–1.082)
Grade 1	106	108.54	83	764.684	1.456 (1.172–1.808)	1.532 (1.232–1.905)
Grade 2	299	340.01	237	697.043	1.299 (1.141–1.480)	1.290 (1.131–1.472)
Grade 3	390	546.81	308	563.272	1.119 (0.998–1.256)	1.096 (0.976–1.230)
Grade 4	399	575.77	317	550.563	1.124 (1.004–1.259)	1.118 (0.998–1.253)
Grade 5	558	896.54	421	469.581	0.994 (0.900–1.097)	0.968 (0.876–1.069)
Grade 6	551	860.55	440	511.302	1.051 (0.954–1.159)	0.996 (0.904–1.098)
By disability type						
People without disabilities					1	1
Physical						
Grade 1–3	256	361.461726	205	567.142	1.121 (0.975–1.289)	1.188 (1.032–1.367)
Grade 4–6	901	1444.725425	693	479.676	0.997 (0.921–1.078)	1.010 (0.933–1.093)
Communication						
Grade 1–3	175	208.57	145	695.194	1.299 (1.102–1.532)	1.176 (0.997–1.388)
Grade 4–6	489	706.33	390	552.153	1.135 (1.024–1.258)	1.042 (0.939–1.155)
Brain						
Grade 1–3	214	217.86	167	766.530	1.448 (1.242–1.689)	1.453 (1.243–1.698)
Grade 4–6	105	154.76	84	542.782	1.152 (0.929–1.429)	0.966 (0.778–1.201)
Mental						
Grade 1–3	44	59.89	33	551.056	1.123 (0.798–1.582)	1.369 (0.966–1.940)
Grade 4–6	NA	NA	NA	NA	NA	NA
Internal organ						
Grade 1–3	106	147.57	78	528.569	1.031 (0.825–1.290)	0.921 (0.735–1.153)
Grade 4–6	13	27.06	11	406.544	0.903 (0.500–1.632)	0.862 (0.476–1.560)

Adjusted for age, sex, income, endemic area, Charlson comorbidity index, seer stage.

**Table 7:** Cancer-specific mortality in female patients diagnosed with bile duct cancer.

	N	Duration	No. of Death	Rate Per 1000	Crude HR	Adjusted HR 2
By disability						
People without disabilities	4203	6595.73	3295	499.566	1	1
People with disability	1390	2064.58	1085	525.531	1.028 (0.959–1.101)	1.016 (0.948–1.089)
By disability grade						
People without disabilities					1	1
Grade 1–3	412	476.94	339	710.787	1.277 (1.142–1.428)	1.260 (1.125–1.410)
Grade 4–6	978	1587.64	746	469.879	0.944 (0.872–1.022)	0.934 (0.863–1.012)
Grade 1	68	61.33	59	962.005	1.566 (1.210–2.026)	1.657 (1.279–2.148)
Grade 2	171	201.41	140	695.100	1.219 (1.029–1.444)	1.196 (1.009–1.417)
Grade 3	173	214.19	140	653.608	1.239 (1.046–1.468)	1.203 (1.016–1.426)
Grade 4	317	485.35	243	500.669	0.976 (0.857–1.112)	0.915 (0.803–1.043)
Grade 5	396	697.27	294	421.647	0.874 (0.776–0.985)	0.878 (0.779–0.990)
Grade 6	265	405.02	209	516.018	1.018 (0.885–1.171)	1.058 (0.919–1.217)
By disability type						
People without disabilities					1	1
Physical						
Grade 1–3	109	144.31	87	602.885	1.151 (0.931–1.425)	1.165 (0.941–1.442)
Grade 4–6	687	1215.87	508	417.809	0.856 (0.780–0.940)	0.864 (0.787–0.950)
Communication						
Grade 1–3	123	143.45	99	690.152	1.232 (1.009–1.505)	1.135 (0.928–1.387)
Grade 4–6	259	336.52	212	629.973	1.203 (1.047–1.382)	1.120 (0.974–1.288)
Brain						
Grade 1–3	122	128.88	106	822.446	1.445 (1.191–1.754)	1.374 (1.131–1.671)
Grade 4–6	28	28.78	22	764.510	1.239 (0.814–1.884)	1.144 (0.751–1.742)
Mental						
Grade 1–3	28	28.86	24	831.567	1.357 (0.908–2.027)	1.909 (1.272–2.864)
Grade 4–6	NA	NA	NA	NA	NA	NA
Internal organ						
Grade 1–3	30	31.44	23	731.599	1.241 (0.824–1.871)	1.348 (0.893–2.035)
Grade 4–6	4	6.48	4	617.665	1.260 (0.472–3.359)	1.973 (0.739–5.271)

Adjusted for age, sex, income, endemic area, Charlson comorbidity index, seer stage.

## Data Availability

Not applicable.

## Supplementary Materials

The following supporting information can be downloaded at: https://www.mdpi.com/article/10.3390/ijerph192416625/s1, Figure S1: Comparison of cancer-specific survival by disability severity; Table S1: Disability categories; Table S2: Baseline characteristics of the target population; Table S3-1: Overall mortality in patients with bile duct cancer; Table S3-2: Overall mortality in men patients diagnosed with bile duct cancer; Table S3-3: Overall mortality in female patients diagnosed with bile duct cancer.
