# Supplementary material for: Disparities in the Diagnosis and Treatment of Bile Duct Cancer in People with Disabilities: A National Cohort Study in South Korea
Source: Int J Environ Res Public Health. 2022 Dec 10;19(24):16625. doi: 10.3390/ijerph192416625 (PMC9779117; doi:10.3390/ijerph192416625)
Supplement: Supplementary file 1 [file ijerph-19-16625-s001.zip › ijerph-2035036-supplementary.pdf]

Supplementary Table S1. Disability categories.

Category	Type of disability
Physical	amputation, motor disturbance, joint disability, disfigurements or deformities, spinal cord injury, facial, epilepsy
Brain	brain impairment
Communication	sight, hearing or speech problems
Mental	difficulty learning or understanding things, mental illness
Internal organ	functional impairment of internal organ diseases such as kidney, heart, liver, lung, intestine

Supplementary Table S2. Baseline characteristics of the target population.

		All cohort	People without disabilities	People with disabilities	Severity		Type				
					Severe	Mild	Physical	Communication	Brain	Mental	Internal organ
		N (%)	N (%)	N (%)	N (%)	N (%)	N (%)	N (%)	N (%)	N (%)	N (%)
		11,105,800(100.0)	8,329,350(75.0)	2,776,450(25.0)	1,091,794(39.3)	1,684,656(60.7)	1,436,219(51.7)	585,986 (21.1)	307,026 (11.1)	307,720 (11.1)	139,499 (5.0)
**Age**	<65	7,495,161(67.4)	5,638,970(67.7)	1,856,191(66.9)	785,173(71.9)	1,071,018(63.6)	942,124 (65.6)	303,605 (51.8)	151,871 (49.5)	294,498 (95.7)	89,911 (64.5)
	≥65	3,610,639(32.6)	2,690,380(32.3)	920,259(33.1)	306,621(28.1)	613,638(36.4)	494,095 (34.4)	282,381 (48.2)	155,155 (50.5)	13,222 (4.3)	49,588 (35.5)
**Sex**	Male	6,498,991(58.5)	4,874,242(58.5)	1,624,749(58.5)	653,958(59.9)	970,790(57.6)	837,697 (58.3)	340,738 (58.1)	176,904 (57.6)	182,319 (59.2)	87,091 (62.4)
	Female	4,606,809(41.5)	3,455,108(41.5)	1,151,701(41.5)	437,836(40.1)	713,866(42.4)	598,522 (41.7)	245,248 (41.9)	130,122 (42.4)	125,401 (40.8)	52,408 (37.6)
**Income**	Below poverty line (lowest)	791,358(7.1)	323,466(3.9)	467,892(16.9)	306,705(28.1)	161,187(9.6)	175,603 (12.2)	75,881 (12.9)	52,792 (17.2)	140,984 (45.8)	22,630 (16.2)
	I	2,281,035(20.5)	1,759,103(21.1)	521,932(18.8)	177,224(16.2)	344,708(20.5)	288,625 (20.1)	115,371 (19.7)	50,367 (16.4)	43,264 (14.1)	24,305 (17.4)
	II	1,923,181(17.3)	1,459,618(17.5)	463,563(16.7)	157,095(14.4)	306,468(18.2)	258,990 (18.0)	101,167 (17.3)	46,185 (15.0)	35,045 (11.4)	22,176 (15.9)
	III	2,505,208(22.6)	1,929,680(23.2)	575,528(20.7)	197,495(18.1)	378,033(22.4)	317,603 (22.1)	124,440 (21.2)	63,392 (20.6)	40,752 (13.2)	29,341 (21.0)
	IV (highest)	3,605,018(32.5)	2,857,483(34.3)	747,535(26.9)	253,275(23.2)	494,260(29.3)	395,398 (27.5)	169,126 (28.9)	94,288 (30.7)	47,675 (15.5)	41,047 (29.4)

Supplementary Table S3-1. Overall mortality in patients with bile duct cancer.

	N	Duration	No. of death	rate per 1000	Crude HR	Adjusted HR 1	Adjusted HR 2
By disability							
People without disabilities	11372	18189.09	9524	523.61	1	1	1
People with disability	3693	5392.79	3176	588.934	1.097(1.054-1.142)	1.083(1.040-1.128)	1.074(1.031-1.119)
By disability grade							
People without disabilities					1	1	1
Grade 1-3	1207	1472.29	1070	726.759	1.281 1.202 1.364	1.282(1.203-1.366)	1.266(1.188-1.350)
Grade 4-6	2486	3920.51	2106	537.175	1.023 0.976 1.073	1.005(0.958-1.053)	0.999(0.953-1.047)
Grade 1	174	169.87	158	930.113	1.569(1.340-1.836)	1.629(1.392-1.907)	1.648(1.407-1.930)
Grade 2	470	541.42	420	775.741	1.324(1.201-1.460)	1.318(1.195-1.454)	1.309(1.186-1.444)
Grade 3	563	761.00	492	646.517	1.178(1.076-1.289)	1.174(1.072-1.286)	1.151(1.051-1.261)
Grade 4	716	1061.13	616	580.516	1.083(0.998-1.175)	1.022(0.942-1.110)	1.044(0.962-1.133)
Grade 5	954	1593.81	794	498.177	0.970(0.902-1.043)	0.948(0.882-1.020)	0.954(0.887-1.026)
Grade 6	816	1265.57	696	549.948	1.037(0.960-1.120)	1.060(0.982-1.145)	1.015(0.940-1.097)
By disability type							
People without disabilities					1	1	1
Physical							
Grade 1-3	365	505.77	310	612.9	1.114(0.995-1.248)	1.184(1.057-1.326)	1.173(1.047-1.314)
Grade 4-6	1588	2660.59	1317	495.0	0.951(0.898-1.008)	0.963(0.909-1.02)	0.963(0.908-1.02)
Communication							
Grade 1-3	298	352.02	267	758.48	1.305(1.155-1.474)	1.182(1.046-1.335)	1.180(1.045-1.333)
Grade 4-6	748	1042.85	656	629.05	1.173(1.083-1.269)	1.081(0.999-1.170)	1.077(0.995-1.166)
Brain							
Grade 1-3	336	346.74	309	491.14	1.536(1.371-1.720)	1.474(1.315-1.652)	1.502(1.340-1.684)
Grade 4-6	133	183.54	118	642.93	1.204(1.004-1.444)	1.139(0.949-1.367)	1.032(0.859-1.238)
Mental							

Grade 1-3	72	88.74	64	721.16	1.273(0.996-1.628)	1.655(1.293-2.118)	1.653(1.289-2.122)
Grade 4-6	-	-	-		-	-	-
Internal organ							
Grade 1-3	136	179.01	120	670.37	1.184(0.989-1.418)	1.221(1.019-1.463)	1.100(0.917-1.318)
Grade 4-6	17	33.53	15	447.32	0.898(0.541-1.490)	0.839(0.505-1.393)	0.920(0.554-1.528)

Adjusted HR 1: age, sex, Charlson comorbidity

Adjusted HR 2: 1+ income, endemic area, seer stage

Supplementary Table S3-2. Overall mortality in men patients diagnosed with bile duct cancer.

	N	Duration	No. of death	rate per 1000	Crude HR	Adjusted HR 1	Adjusted HR 2
By disability							
People without disabilities	7169	11593.37	5995	517.106	1	1	1
People with disability	2303	3328.22	1994	599.119	1.128(1.072-1.187)	1.117(1.062-1.176)	1.095(1.040-1.153)
By disability grade							
People without disabilities					1	1	1
Grade 1-3	795	995.35	704	707.286	1.273(1.178-1.377)	1.283(1.186-1.388)	1.257(1.161-1.362)
Grade 4-6	1508	2332.87	1290	552.968	1.062(1.000-1.128)	1.045(0.984-1.110)	1.025(0.965-1.089)
Grade 1	106	108.54	94	866.028	1.535(1.252-1.882)	1.598(1.303-1.960)	1.606(1.308-1.971)
Grade 2	299	340.01	268	788.217	1.370(1.212-1.548)	1.382(1.222-1.562)	1.357(1.199-1.537)
Grade 3	390	546.81	342	625.451	1.155(1.036-1.288)	1.156(1.036-1.289)	1.128(1.011-1.259)
Grade 4	399	575.77	351	609.614	1.153(1.035-1.284)	1.109(0.996-1.235)	1.138(1.022-1.268)
Grade 5	558	896.54	466	519.774	1.015(0.924-1.116)	0.983(0.895-1.081)	0.984(0.895-1.082)
Grade 6	551	860.55	473	549.649	1.049(0.955-1.152)	1.065(0.970-1.170)	0.993(0.904-1.091)
By disability type							
People without disabilities					1	1	1
Physical							
Grade 1-3	256	361.46	219	605.873	1.113(0.973-1.274)	1.211(1.058-1.387)	1.18(1.03-1.352)
Grade 4-6	901	1444.73	758	524.667	1.008(0.935-1.088)	1.038(0.963-1.12)	1.02(0.946-1.101)
Communication							
Grade 1-3	175	208.57	157	752.73	1.313(1.120-1.538)	1.158(0.988-1.357)	1.182(1.008-1.386)
Grade 4-6	489	706.33	429	607.37	1.158(1.050-1.277)	1.063(0.964-1.173)	1.057(0.958-1.166)
Brain							
Grade 1-3	214	217.86	196	899.64	1.584(1.374-1.827)	1.560(1.352-1.800)	1.580(1.368-1.824)
Grade 4-6	105	154.76	92	594.48	1.166(0.949-1.433)	1.073(0.872-1.319)	0.964(0.784-1.186)
Mental							

Grade 1-3	44	59.89	38	634.55	1.199(0.872-1.650)	1.539(1.118-2.120)	1.482(1.071-2.051)
Grade 4-6	-	-	-	-	-	-	-
Internal organ							
Grade 1-3	106	147.57	94	636.99	1.158(0.945-1.420)	1.148(0.935-1.409)	1.027(0.836-1.261)
Grade 4-6	13	27.06	11	406.54	0.832(0.461-1.504)	0.728(0.403-1.317)	0.777(0.430-1.407)

Adjusted HR 1: age, sex, Charlson comorbidity

Adjusted HR 2: 1+ income, endemic area, seer stage

Supplementary Table S3-3. Overall mortality in female patients diagnosed with bile duct cancer.

	N	Duration	No. of death	rate per 1000	Crude HR	Adjusted HR 1	Adjusted HR 2
By disability							
People without disabilities	4203	6595.73	3529	535.043	1	1	1
People with disability	1390	2064.58	1182	572.514	1.048(0.981-1.119)	1.028(0.962-1.098)	1.032(0.973-1.106)
By disability grade							
People without disabilities							
Grade 1-3	412	476.94	366	767.399	1.298(1.165-1.445)	1.271(1.141-1.416)	1.275(1.144-1.421)
Grade 4-6	978	1587.64	816	513.970	0.964(0.893-1.040)	0.947(0.877-1.022)	0.954(0.884-1.030)
Grade 1	68	61.33	64	1043.53	1.608(1.256-2.060)	1.661(1.296-2.128)	1.691(1.318-2.169)
Grade 2	171	201.41	152	754.68	1.248(1.060-1.468)	1.207(1.025-1.420)	1.215(1.032-1.430)
Grade 3	173	214.19	150	700.29	1.245(1.057-1.466)	1.216(1.032-1.433)	1.210(1.027-1.425)
Grade 4	317	485.35	265	546.00	0.995(0.878-1.127)	0.924(0.815-1.047)	0.930(0.821-1.055)
Grade 5	396	697.27	328	470.41	0.909(0.812-1.018)	0.902(0.805-1.010)	0.913(0.815-1.023)
Grade 6	265	405.02	223	550.58	1.016(0.888-1.164)	1.056(0.922-1.210)	1.059(0.924-1.213)
By disability type							
People without disabilities							
Physical							
Grade 1-3	109	144.31	91	630.603	1.129(0.917-1.391)	1.113(0.904-1.371)	1.141(0.926-1.406)
Grade 4-6	687	1215.87	559	459.754	0.879(0.804-0.961)	0.875(0.800-0.957)	0.888(0.811-0.971)
Communication							
Grade 1-3	123	143.45	110	766.836	1.289(1.066-1.559)	1.217(1.006-1.471)	1.184(0.979-1.433)
Grade 4-6	259	336.52	227	674.547	1.205(1.053-1.378)	1.125(0.983-1.287)	1.120(0.979-1.282)
Brain							
Grade 1-3	122	128.88	113	876.759	1.452(1.203-1.751)	1.324(1.097-1.598)	1.364(1.129-1.648)
Grade 4-6	28	28.78	26	903.511	1.386(0.942-2.039)	1.429(0.971-2.103)	1.285(0.873-1.892)
Mental							
Grade 1-3	28	28.86	26	900.865	1.391(0.945-2.046)	1.899(1.288-2.799)	1.951(1.320-2.882)

Grade 4-6		-	-	-	-	-	-
Internal organ							
Grade 1-3	30	31.44	26	827.025	1.326(0.901-1.950)	1.577(1.071-2.323)	1.456(0.988-2.146)
Grade 4-6	4	6.48	4	617.665	1.178(0.442-3.140)	1.506(0.565-4.018)	1.831(0.685-4.889)

Adjusted HR 1: age, sex, Charlson comorbidity

Adjusted HR 2: 1+ income, endemic area, seer stage

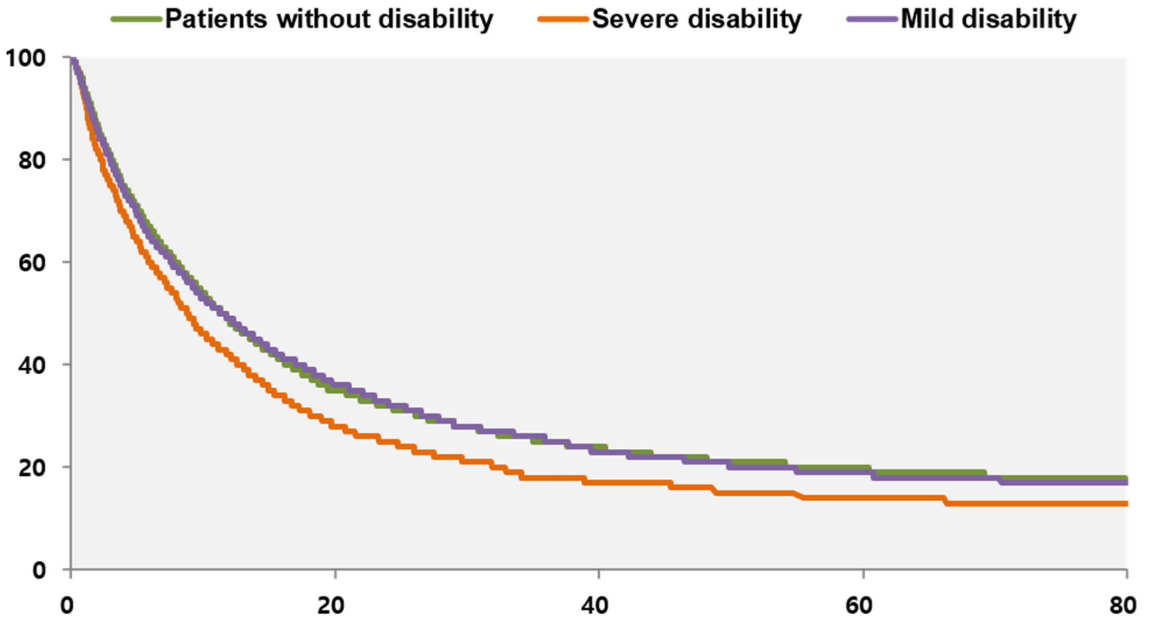

No. at risk					
Patients without disability	11372	3784	2256	1173	430
Severe disability	1207	301	156	72	26
Mild disability	2486	843	471	234	83

	Event N.(%)	Median (95% CI) month
All	11733(77.9)	11.27(10.908-11.663)
Patients without disability	8842(77.8)	11.53(11.138-11.893)
Severe disability	967(80.2)	8.94(8.016-9.593)
Mild disability	1924(77.4)	11.50(10.448-12.485)

Supplementary Figure S1. Comparison of cancer-specific survival by disability severity.
